# Anæsthesia and Anæsthetics

**Published:** 1879-07

**Authors:** J. J. Caldwell

**Affiliations:** Baltimore, Md.


### ANÆSTHESIA AND ANÆSTHETICS.

BY DR. J. J. CALDWELL, OF BALTIMORE, MD.

*Read before the Dental Association of the State of Maryland and the District of Columbia.*

*Gentlemen:*—At the session of 1877, at Oakland, you honored me with the appointment of chairman of this section. This season that honor has been conferred upon our brother,

H. C. Thompson, D. D. S., but, unfortunately, owing to affliction and ill-health, he has been unable to comply. While I thoroughly appreciate the fact that I will not be able to handle the subject with that masterly skill as he, still I will endeavor to present it in a plain, practical manner, furnishing such advanced data as the present status of our science will admit.

Owing to professional engagements last year, I was unable to act as chairman of this section, and Dr. H. C. Thompson kindly prepared and read the paper on the subject before us. A brief review of that paper will more readily lead us into the subject-matter. If you remember, he first considered the different modes of investigation; the physiological and pathological conditions of the body while under the anæsthetic influence; the various experiments on animals of the lower order under its influence; the necessity of the administration of stimulants previous to the inhalation of these gases; the effects upon the circulation, then upon the nervous centers; sudden death by heart-paralysis, and the tests for the purity and impurity of anæsthetics; the action upon the eye, dilatation and contraction of the pupil; the value of these phenomena, and the value of the different restoratives; such as electricity, pure oxygen, Dr. Dabney's remedy, nitrite of amyl; Nelaton's remedy of standing on the head; Marshall Hall's ready method, etc.

The next subject considered was the use of the spectroscope on the blood changes produced by anæsthetics, ably handled by the distinguished spectroscopist of this country, Dr. Waterman. So much for the work of last year.

As to whom the great honor should be awarded of having discovered such a valuable and benign agency as anæsthesia, we can not but coincide with the opinion pronounced by Dr. J. Marion Sims, in the Virginia Medical Monthly, of May, 1877, that the distinguished individual was Dr. Crawford W. Long, of Athens, Georgia, who extirpated a tumor from the neck of James W. Venable, of Jefferson, Jackson County, Georgia, March 30. 1842, while he was completely anæsthetized by the inhalation of sulphuric ether.

As remarked, however, by Dr. Sims, "to each claimant for the honor of discovering anæsthesia is due a certain measure of credit." The names of Long, Wells, Morton and Jackson will doubtless be associated together as co-laborers in the great work, and to these must be added the immortal name of Sir James Y. Simpson, who introduced chloroform and enlarged the domain of anæsthesia.

Dr. Wellford remarks that in childbirth and obstetrics the use of chloroform in careful and experienced hands, is as nearly safe as any other article of the materia medica of equal potency and benefit. In obstetrics, two million cases have been reported, in which it has been successfully used, and medical journals have reported only four or five cases of death, and these were not under the management of medical men. Undoubtedly the chief danger arising from the use of chloroform is the impurity of the article. Methylic chloroform, which is liable to contain six per cent. of a dangerous empyreumatic oil, has, as surmised by Dr. Wellford, caused a large proportion of the deaths by its inhalation, particularly in the hands of English surgeons, as a very large quantity of such chloroform is prepared and sold in England. The pharmacopia gives a formula for chloroform purification, which is the only safe one to use. Another source of danger is the improper administration of chloroform. "To produce ethereal anæsthesia," says Dr. Wellford, "it is absolutely necessary to prevent the entrance of atmospheric air and compel the patient to breathe only pure ethereal vapor. The system is able to exist in the amount of oxygen thus obtained, and, consequently, one is compelled to use large quantities of the article. If you were to pursue the same plan with chloroform you would inevitably produce the death of your patient by suffocation or pure asphyxia, and all the more readily because he was somewhat anæsthetized, and was, therefore, unable to make the necessary effort to obtain relief; consequently, any one accustomed to the use of ether should be aware of the difference of application, or he might unwittingly cause a fatal result." "Too frequently, the operator, anxious to save time, places the sponge too close to the patient's nose, causing a fatal result by suffocation, only hastened but not caused by the chloroform." Dr. Marion Sims, with others, has expressed the opinion that "unfavorable effects of the remedy were primarily produced by anæmia of the brain." But Anstie remarks that "two distinct lines of narcotic impressions may be traced in the action of chloroform upon the animal organism; the development of the one or the other depending entirely on the rapidity with which the arterial circulation becomes charged with the narcotic agent. When the impregnation of the blood takes place with moderate rapidity, the sympathetic system is the ultimum moreius, and death begins at the lungs. When, on the contrary, the circulation becomes rapidly charged with a large proportion of chloroform, the narcotic effect may fall with such force on the sympathetic nerves as to extinguish their vitality. The greatest possible importance is attached to this distinction, for one of the causes of the latter occurrence is the production of instantaneous paralysis of the heart."

Says Anstie:

"1. The effect upon the intellectual operations during the first few seconds of inhalation appears to be an invigorating one. Needless timidity and distress vanish while consciousness is as yet unaffected, and the expression of the face is bright and intelligent. Before the conclusion of the first minute, however, the approach of intellectual paralysis is perceived in the great majority of cases; the patient, if narrowly observed, will be found to be losing the consciousness of actual events, and, in many instances, this state is accompanied by a disposition to voluble talking of a more less unreasonable character. As the affection increases, the very curious phenomena of narcotic reminiscence make their appearance, if at all. This never happens till inhalation has proceeded for at least a minute, and, usually, longer. Paralysis of the tongue usually prevents the utterance of connected language in a few seconds more, but the patient will sometimes continue to babble in a way indicating the existence of consciousness till a very advanced stage of anæsthesia; and, in cases where it is not necessary to carry narcotism to its furthest, this may go on throughout the maintenance of anæsthesia. Signs of emotional disturbance sometimes accompany the first diminution of consciousness; the patient is angry, or pathetic, or sentimental, and gives expression to these feelings in words, with no reference, or only a confused reference, to persons and things actually present.

"2. The power to co-ordinate voluntary movement is usually affected very soon after the first impairment of consciousness; the patient makes vain efforts to grasp objects, and very soon afterward his articulation becomes slightly impaired.

"3. Sensation and voluntary motion. These functions, although their alteration may be said, on the whole, to proceed pari passu, both of them being earliest disturbed in the lower limbs (as was observed of alcohol) must be separately reported on.

"(a) The first effect on sensation which is noticeable is the removal or mitigation of any pain from which the patient suffers; this is often effected by one or two inspirations only. Evidence of paralysis of skin sensibility. Before the end of the second minute, however, there was very considerable paralysis of the whole skin-surface. The conjunctiva always retained sensibility later than the skin, with certain exceptions presently to be noted. In the great majorrity of instances, however, it was rendered insensitive by the time that contraction of the pupil was well marked, (third stage). Certain portions of the skin and subcutaneous tissue, however, retain their sensibility with extraordinary tenacity; these are the matrix of the great toe-nail, the margin of the anus, and the whole of the skin of the organs of generation. It is impossible to obliterate their sensibility without pushing chloroformization to a degree which greatly surpasses that required for ordinary purposes. This observation is confirmed by experience with animals, and its importance can not be too highly estimated, for it explains the frequency with which death has happened in the course of anæsthesia induced for the performance of operations for phimosis, evulsion of the toe-nails, hemorrhoids, etc. All kinds of fanciful reasons have been given for this fatality of chloroform in such trifling operations, but there is no doubt that this is the true one.

"(b). Voluntary motor power is usually not much diminished during the first minute of inhalation, and the patient occasionally struggles from alarm, but in the case of regularly induced anæsthesia, this voluntary struggling is limited in a great majority of instances, to this early stage; and simple paralysis, gradually increasing, is the only further phenomenon connected with the voluntary muscles. This paralysis had reached a high degree by the end of the second minute.

"4. Under the head of 'Effects on the Sympathetic System,' are classed: (a) Flushing of the face accompanied with heat (and sometimes with sweating,) and not fairly attributable to the heat of the room or to the effect of struggling, etc., and is always a late phenomenon, unlike the case of alcohol and ether, in which sympathetic paralysis is one of the earliest symptoms. (b) Contraction and dilatation of the pupil. While contraction occurs in the majority of cases by the end of the second minute, and is fully developed during the third minute, dilatation rarely occurs till the latest stages, and may fairly be considered as a sign that a profound degree of anæsthesia has probably been reached. This observation has been verifiedby many cases. Wide dilatat on of the pupil is very rarely witnessed, except in conjunction with stertorous breathing and considerable depression of the heart's action, both as to force and frequency.

"5. By effects on the medulla oblongata, are meant the production of stertorous breathing, laborious breathing, or both. The phrase is by no means intended to signify mere snoring, but paralysis of the velum palati and buccinators. Laborious breathing—that is, slow and somewhat irregular. Profound modification of the breathing is always accompanied by wide dilatation of the pupil.

"6. 'Reflex' phenomena. (a) Coughing produced. It did not occur in any patient after the conclusion of the second minute, expect as a sequel of vomiting."

Says Anstie again:

"In fifty children, observation was made on the modifications of sensation and voluntary motion, of the functions of the sympathetic system, and the medulla oblongata and on the 'reflex' phenomena. The only matter requiring observation is, that convulsive phenomena were noted in only two cases, and symptoms indicating approaching syncope only in one. The latter fact faithfully reflects the result of my whole chloroform experience, in demonstrating the comparatively small amount of danger to children from weakening of the heart's action under chloroform."

Beard says in his experience children bear chloroform as they do milk.

Anstie says:

"The final observation which has to be recorded as to these one hundred cases is, that the smallest quantity of chloroform employed in any case was two, and the largest three drachms.

"On a review of the observations detailed above, I conclude that—

"I. That the phenomena of anæsthetization of man by chloroform vapor, when this is conducted with method, and is accomplished in a period of about four minutes, are, on the whole, extremely regular.

"II. Under such circumstances the occurrence of any dangerous degree of depression is rare, and may always be remedied by attention.

"III. During a very short period at the commencement of inhalation the vital functions are stimulated, an effect which ceases on the very first occurrence of narcotic symptoms.

"IV. The whole subsequent train of symptoms is that of depression of the various portions of the nervous system in consecutive order of occurrence; the brain being the first lose, and the medulla oblongata and sympathetic system the most tenacious in retaining their proper functions.

"V. The convulsive phenomena occasionally witnessed be long to the category of the paralytic, and not of the stimulant effects of chloroform, unless they are brought about by an accidental local cause.

"VI. Every considerable increase or decrease in the frequency, and every decrease in the force of the respiratory and circulatory movement is a part of the depressing influence of chloroform."

Says Chisholm:

"The only legitimate and rarest of causes of death from anæsthetics now faces us. It is that unknown condition called idiosyncrasy, in which anæsthetics show themselves poisons of extreme activity. The patients who carry about with them this innate fatality exhibit it by no recognized signs. When such persons die from the toxic inhalation, the autopsy reveals absolutely nothing to indicate the destructive effects of the poison.

"Anæsthetics are among the most powerful drugs of the pharmacopia, and must always be administered with caution. To bring a living being to that border land in which life in many respects so simulates death should at no time, be a fool's occupation.

[TO BE CONTINUED.]

